# *Helicobacter pylori* Infection and its Relationship to Metabolic Syndrome: Is it a Myth or Fact?

**DOI:** 10.4103/1319-3767.80377

**Published:** 2011

**Authors:** Waleed I. Albaker

**Affiliations:** Department of Internal Medicine, King Fahad Hospital of the University, Al-Khobar, Kingdom of Saudi Arabia

**Keywords:** Metabolic syndrome, *Helicobactor pylori*, obesity

## Abstract

Metabolic syndrome is one of the most prevalent global health problems that predisposes to Type 2 diabetes. It is strongly linked to insulin resistance, which results in hyperglycemia. Over the past few years, lot of studies have been carried out on *Helicobacter pylori* infection and found a possible causal relationship through releasing some of the interleukins factors, which result in endothelial dysfunction. However, some studies attributed that due to coincidence were not able to establish any causal relationship. In this review, the literature has been reviewed to check this possible association.

The metabolic syndrome (MS) is also known as syndrome X,[1] the insulin resistance syndrome,[2] and the deadly quartet.[3,4] Throughout the years several classifications for the MS have been proposed, emphasizing insulin resistance or visceral obesity. The adult treatment panel III (ATPIII) report three or more of the following diagnostic criteria for MS: (1) Waist circumference >102 cm (male), >88 cm (female). (2) Triglycerides >1.7 mmol/L or drug treatment for high triglycerides. (3) Low HDL: <1.0 mmol/L (male) <1.3 mmol/L (female). (4) Blood pressure >135/85 or on treatment. (5) Fasting blood sugar >5.6 mmol/L or drug treatment for diabetes.[5]

In Saudi Arabia, a study of the prevalence of MS was done in 2005, using the adult treatment panel (ATP) III. It concluded that prevalence of MS is high in Saudi Arabia. The overall age-adjusted prevalence was found to be 39.3%. Age-adjusted prevalence in males is 37.2% while females have a higher prevalence of 42%.[6] This reflects that MS is very prevalent in Saudi community and probably one of the highest worldwide.

*Helicobactor pylori* (HP) is a gram-negative, spiral-shaped pathogenic bacterium that specifically colonizes in the gastric epithelium and causes chronic gastritis, peptic ulcer disease, and/or gastric malignancies.[7,8] The infection induces an acute polymorph nuclear infiltration in the gastric mucosa. If the infection is not effectively cleared, this acute cellular infiltrate is gradually replaced by an immunologically mediated, chronic, predominantly mononuclear cellular infiltrate.[9] The latter is characterized by the local production and systemic diffusion of proinflammatory cytokines,[10] which may exert their effects in remote tissues and organic systems and result in extragastric manifestations.[11]

The prevalence of HP infection varies between countries; generally, the prevalence is about 30% in developed and up to 80% in developing countries.[12] In Saudi Arabia, the prevalence of HP infection markedly increased with age. The prevalence of HP infection rose from 32.4% in those aged 5–10 years to more than 66.4% in those aged 20–30 years and 75% in those over 50 years.[13]

HP infection has been reported to be hyperendemic in Saudi Arabia.[14,15] Reports in the 1990s have shown a prevalence of 68–82.2%[14,15] in various age groups of patients including those with nonulcer dyspepsia.

Diagnosis of HP can be achieved by taking biopsies by endoscopy. However, this procedure is invasive and might not give accurate results if colonization is patchy.[14] For population screening, serodiagnosis remains one of the methods of choice for detecting the prevalence of infection.[15–17] The technique of choice is currently enzyme-linked immunosorbent assay because it is a simple, quick, and low-cost technique that permits immunoglobulin class-specific determinations.[18–25]

Since the discovery of HP, a variety of studies, essentially epidemiological or therapeutic trials, case reports, and others have evaluated the potential direct or indirect involvement of this bacterium in the pathogenesis of various extragastric disorders. Although currently available data do not provide proof of its role in most of them, a potential relationship cannot be ruled out.

Several studies have evaluated the relation of HP infection with coronary artery disease (CAD), a study done by Kowalski concluded that there is a significant link between CAD and infection with HP. Patients infected with CagA-positive HP show significantly greater coronary artery lumen loss and arterial re-stenosis after cardiac catheterization with stent implantation. It showed that HP eradication significantly attenuates the reduction in coronary artery lumen in CAD patients after PTCA, possibly due to the elimination of chronic inflammation and the decline in proinflammatory cytokine release. They also identified the HP deoxyribonucleic acid (DNA) in atherosclerotic plaques of patients with severe CAD and that supported the hypothesis that infection with HP, especially (CagA) positive, may influence the development of atherosclerosis.[26]

The results of another study performed in Korea to evaluate the relationship between HP infection and CAD showed that HP infection has a modest influence on CAD and progressive athermoa. However, further studies are suggested to evaluate this association.[27,28] In June 2010, a study carried out in Italy to evaluate coronary atherosclerotic burden in patients with infection by CagA-positive strains of HP support the association and suggest further studies to better elucidate the mechanism by which CagA-positive strains may promote atherosclerosis.[29] Although these studies showed a positive association, many studies over the last decades have showed no relationship between the two. A study done in Croatia in 2007 showed a higher seroprevalence of HP infection in patients with CAD compared to controls. However, HP seropositivity was not associated with coronary artery risk factors (smoking, high body mass index (BMI), diabetes mellitus (DM), hypertension, total cholesterol, and socioeconomic status) either in the whole study population or in the patients and control subjects analyzed separately.[30] In 2003, a study done in Czech Republic resulted a similar conclusion, and no relationship was found between unstable angina and infection with HP.[31] In 2006, Sotirupoulos and colleagues showed no association of seropositivity to HP with angiographically documented CAD.[32] In Turkey, the relationship between Helicobacter IgG titers and coronary atherosclerosis was evaluated and the researchers concluded that HP IgG titers do not play an important role in the presentation of CAD and do not increase systemic inflammatory response. However, HP IgG antibody titers may correlate with the extent of CAD.[33] In 2004, a study done in Korea revealed that HP infection is not an independent risk factor for coronary heart disease, and it does not alter the coagulation system or evoke the system inflammatory response.[28] Another study done in Poland to detect HP in atherosclerotic plaques did not trace DNA of bacteria in any of the patients that underwent coronary artery bypass graft (CABG).[34]

HP infection may cause dyslipidemia, as it leads to elevated levels of total cholesterol, low-density lipoprotein cholesterol (LDL-c), lipoprotein Lp(a), apolopoprotein apo-B, triglyceride concentrations and decreased levels of high-density lipoprotein cholesterol (HDL-C) and apolipoprotein apoA-1 concentration in the blood.[35,36] In addition, plasma levels of cholesterol and LDL-c were significantly higher in HP positive patients with ischemic stroke compared to HP positive patients.[35,36] It was postulated that chronic HP infection may shift the lipid profile toward an atherogenic direction via the action proinflammatory cytokines, such as interleukins 1 and 6 (IL-1 and IL-6), interferon-alpha (INF-α), and tumor necrosis factor-alpha (TNF-α). These cytokines are capable of affecting lipid metabolism in different ways, including activation of adipose tissue lipoprotein lipase, stimulation of hepatic fatty acid synthesis, and influencing lipolysis.[35,36] The relationship between dyslipidemia and HP eradication is also controversial. After 1 year of eradication of HP in patients with duodenal ulcers, a significant increase of HDL-c, apo-A1, and apo AII levels was observed by Scharnagl and colleagues.[35] Moreover, eradication of HP in healthy subjects seems to increase HDL-c and decrease LDL-c level.[35] Also, 6 months following successful eradication of HP infection, the plasma levels of total cholesterol and LDL-c were found to be significantly lower than those in *H. pylori* positive controls and HP positive patients with stroke.[35]

The circulating levels of the two hormones involved in body weight and food intake, ghrelin, and leptin, in relation to HP status in adult males have been investigated. In particular, based on several studies, Roper *et al.*[36] hypothesized that gastric HP colonization reduced circulating levels of leptin and ghrelinand he examined the gastric juice levels of leptin and ghrelin in well-defined fasting HP-positive and HP-negative adult male subjects. The results showed that HP colonization was associated with reduced circulating leptin levels, independent of BMI, and fundic ghrelin and leptin levels were directly related. Levels of ghrelin in HP-positive and -negative patients were similar. Ghrelin was present in gastric juice over a large range of concentration, and was strongly correlated with gastric pH. A similar study has been conducted by Pacifico *et al*.. on prepubertal children population.[37] They evaluated the relation between circulating ghrelin and leptin concentrations and HP infection with the levels of these hormones after eradication treatment and body composition in prepubertal children. Interestingly, the results showed that serum ghrelin concentration is inversely related to the severity of HP-associated gastritis. In these youngsters, long-term eradication of HP infection was associated with a significant increase of BMI, lean, and fat mass along with a significant decrease in circulating ghrelin levels and an increase in leptin levels.

Many authors reported high prevalence of HP infection among patients with DM Type 2, suggesting that delayed gastric clearance could be attributed to bacterial colonization or overgrowth in the gastrointestinal tract as a result of autonomic neuropathy. In contrast, some studies reported a lower prevalence of HP in diabetic patients compared to healthy population. This issue has been investigated by Hamed and colleagues.[38] They evaluated in a very interesting study, the prevalence of HP infection in patients with DM, as well as the association between diabetic vascular complications and HP infection. They found that the influence of HP was higher in patients with DM compared to healthy controls. Carotid artery intima-media thickness was significantly higher in HP-infected patients. Inflammatory biomarkers, as interleukin (IL)-6, and tumor necrosis factor-alpha (TNF-α), were significantly associated with HP infection.

HP infection and its contribution to atherosclerosis and cardiovascular diseases are still controversial. Hence, insulin resistance is the pathophysiologic background of the clinical features of atherosclerosis and cardiovascular diseases. Some studies performed to examine the association between HP infection and insulin resistance. Yoshikawa *et al.*[39] evaluated the involvement of either HP infection or impaired glucose (IG) metabolism in increasing of “brachial-ankle pulse wave velocity” (baPWV), a well known indicator of arterial stiffness and severity of vascular damage. In particular, HP seropositivity has been demonstrated to be a potential risk factor for increased baPWV levels, and to accelerate the effect of IG on increasing of baPWV, especially in younger subjects.

There are contradictory reports on HP prevalence and its relationship to late complications of DM. A study by Bener *et al.*[40] suggested that there is a significant association between HP infection and Type 2 DM and, interestingly, this infection is significantly higher in diabetic obese patients. On the contrary, Demir *et al.*[41] showed that the prevalence of HP infection did not differ significantly between diabetic patients and nondiabetic controls. However, HP-infected diabetic patients had a significantly higher incidence of neuropathy.

The relationship between DM and HP infection is controversial. According to some studies, there is a high prevalence of HP infection in patients with either Type 1 or Type 2 DM which is correlated with the duration of DM, the presence of dyspeptic symptoms, cardiovascular autonomic neuropathy, age, gender, BMI, blood pressure, fasting glucose, and the glycated hemoglobin levels

(HbA1c).[40–49] In particular, the prevalence of HP infection was found to be high in obese middle-aged female patients with a long-standing DM, dyspeptic symptoms, cardiovascular autonomic neuropathy, and increased blood pressure, fasting glucose levels, and HbA1c values.[40–49] This could be related to a reduced gastric motility and peristaltic activity, various chemical changes in gastric mucosa following nonenzymatic glycosylation processes and an impaired nonspecific immunity observed in diabetic patients.[40–49] In contrast, other studies showed that HP infection is not associated with DM, as the absence of microangiopathy in patients with DM may be a negative factor for colonization by HP. Because microvascular changes in the gastric mucosa may create an unfavourable environment for the establishment or survival of HP.[40–49]

HP infection is another issue which is contentious and deserves further investigation, as only few data are available. According to some data, there is no relationship between HP infection and diabetic complications, such as nephropathy, retinopathy, and/or microangiopathy while other data showed that virulent strains of HP, such as cytotoxin-associated gene CagA+, are associated with macroangiopathy, neuropathy, and microalbuminuria in Type 2 diabetic patients. This may be due to an immune-mediated injury at the level of the endothelium caused by a systemic immune response to the infection, leading to albumin leakage.[41,50]

A Japanese study in 2009 included a large population of 1598 asymptomatic subjects who had been evaluated, and the results came out to be supportive of the significant and independent contribution of HP promoting insulin resistance in a large asymptomatic population.[51] Another study from China in 2009 have shown that HP infection has a significant effect on the daily blood glucose level and blood glucose fluctuation in the patients with Type 2 diabetes.[52] The effect of HP has been studied in relation to the lipid profile, but there has been a controversy. In a study from Congo, certain components of the MS/insulin resistance, gender, cardiovascular diseases, and HP seropositivity infection were analyzed and the response of these cardiovascular risk factors to HP titers after an antibiotic course was evaluated. The results showed evidence supporting the association of seropositivity to HP with cardiovascular disease and elevated number of components of MS. *Helicobacter pylori* infection might generate atherosclerosis or MS; particularly in men with seropositive HP. *Helicobacter pylori* infection might be one of the risk factors of atherosclerosis thorough inflammation (fibrinogen) and modulation of glucose and lipid profiles.[53] The results of another Japanese cross-sectional study comprising of 5488 men and 1906 women showed that HP seropositivity increased significantly with age, both in men and women. *Helicobacter pylori* seropositivity was significantly higher in cases with MS compared with those without MS.[54] Similar results were reported from Iran in which MS was found to occur very frequently in the general population, and had a significant association with prior infection with Chlamydia pneumonia, HP, cytomegalovirus, and herpes simplex virus Type 1.[55]

The recent study from Turkey evaluated the association between HP and MS analyzing the effect of HP eradication on insulin resistance, serum lipids, and low-grade inflammation.[56] His study showed beneficial effects of HP eradication on insulin resistance, atherogenic lipid abnormalities, and low-grade inflammation. The results suggested that HP eradication may prevent CAD and MS.[56]

## CONCLUSION

The association of MS and *H. pylori* is still controversial with emphasis on the possible linkage between them. However, the high prevalence of both MS and HP infection might explain the coincidence.
